# Scaling photosynthetic function and CO_2_ dynamics from leaf to canopy level for maize – dataset combining diurnal and seasonal measurements of vegetation fluorescence, reflectance and vegetation indices with canopy gross ecosystem productivity

**DOI:** 10.1016/j.dib.2021.107600

**Published:** 2021-11-20

**Authors:** Petya Campbell, Elizabeth Middleton, Karl Huemmrich, Lauren Ward, Tommaso Julitta, Peiqi Yang, Christiaan van der Tol, Craig Daughtry, Andrew Russ, Joseph Alfieri, William Kustas

**Affiliations:** a University of Maryland Baltimore County, MD, USA; b NASA Goddard Space and Flight Center, Greenbelt, MD, USA; c University of Hawai'i at Mañoa, Hawai'i, USA; d J-B Hyperspectral Devices GmbH, Dusseldorf, Germany; e University of Twente, Twente, the Netherland; f USDA Agricultural Research Center, Beltsville, MD, USA

**Keywords:** Vegetation, Maize, Leaf-level chlorophyll fluorescence, Canopy solar induced fluorescence (SIF), Reflectance, Photosynthetic function, Eddy covariance observations, Gross Ecosystem Productivity (GEP)

## Abstract

Recent advances in leaf fluorescence measurements and canopy proximal remote sensing currently enable the non-destructive collection of rich diurnal and seasonal time series, which are required for monitoring vegetation function at the temporal and spatial scales relevant to the natural dynamics of photosynthesis. Remote sensing assessments of vegetation function have traditionally used actively excited foliar chlorophyll fluorescence measurements, canopy optical reflectance data and vegetation indices (VIs), and only recently passive solar induced chlorophyll fluorescence (SIF) measurements. In general, reflectance data are more sensitive to the seasonal variations in canopy chlorophyll content and foliar biomass, while fluorescence observations more closely relate to the dynamic changes in plant photosynthetic function. With this dataset we link leaf level actively excited chlorophyll fluorescence, canopy proximal reflectance and SIF, with eddy covariance measurements of gross ecosystem productivity (GEP). The dataset was collected during the 2017 growing season on maize, using three automated systems (i.e., Monitoring Pulse-Amplitude-Modulation fluorimeter, Moni-PAM; Fluorescence Box, FloX; and from eddy covariance tower). The data were quality checked, filtered and collated to a common 30 minutes timestep. We derived vegetation indices related to canopy functioning (e.g., Photochemical Reflectance Index, PRI; Normalized Difference Vegetation Index, NDVI; Chlorophyll Red-edge, Clre) to investigate how SIF and VIs can be coupled for monitoring vegetation photosynthesis. The raw datasets and the filtered and collated data are provided to enable new processing and analyses.

## Specifications Table

**Table:** 

Subject	Biology, Agricultural Science, Ecology, Plant Physiology, Remote Sensing, Vegetation Traits
Specific subject area	Remote sensing monitoring of vegetation photosynthetic function, leaf and canopy reflectance and fluorescence measurements
Type of data	Tables of contemporaneous, coupled time series of observations
How data were acquired	Actively excited chlorophyll fluorescence measurements were collected using the Pulse Amplitude Modulated (PAM) approach, on two shaded and three sunlit leaves. The measurements were collected using an automated MoniPAM Data Acquisition system (MONI-DA, Heinz Walz GmbH, Effeltrich, Germany^1^) outfitted with five emitter-detector probes. Canopy upwelling and down-welling passive optical measurements were collected using a Dual FLuorescence boX (FloX, JB Hyperspectral Devices UG, Dusseldorf, Germany) [1] system. These spectral measurements were processed to provide reflectance and solar induced fluorescence (SIF) using two open-source R packages [2,3] (i.e., FieldSpectroscopyDP and FieldSpectroscopyCC), available at: https://github.com/tommasojulitta/FieldSpectroscopyDP and https://github.com/tommasojulitta/FieldSpectroscopyCC.
Data format	The dataset includes tables of the following data types and formats: • Raw data: instrument readings and calibration files (formatted as digital numbers, comma delimited (CSV) files, American Standard Code for Information Interchange (ASCII) files) for use by researchers closely familiar with the instrumentation. The names of the raw datasets/files are: 2017_OPE3_Flux_data_raw.xls; MoniPAM2017_all.xls and MoniPAM_OPE3_2017_WinControl3.zip; FloX_DataOPE3_2017_raw.zip • Filtered, coupled and collated datasets, appended with metadata, in Excel format described in Table 1. The filenames are: 2017OPE3_moni_FLOX_FLUX.xlsx and FLoX_R_OE3_2017all.xlsx.
Parameters for data collection	Vegetation growth stages of the corresponding datasets (days of year, DOY): Data were collected throughout the growing season, to represent the following growth stages DOY 192-209 corn young (Yn), DOY 220-235 mature (M), DOY 245-247 early senescence (S1) and DOY 255-257 advanced senescence (S2). Daytime periods: Data were collected continuously, covering the following daylight periods - morning (8:45-10:45), mid-day (11:45-13:15), afternoon (15:15-16:45) Moni-PAM measurements: We collected leaf actively induced florescence measurements using five emitter-detector probes. Three probes (IDs: CFME0262A, CFME0263, CFME0265A) were used for sunlit leaf measurements and two probes (IDs: CFME0264A, CFME0335A) for measuring shaded leaves. The processed and curated dataset is provided using the local Eastern Daylight Time (EDT).
Description of data collection	All data were collected under natural growing conditions in the field (i.e., *in situ*) at the OPE3 site on maize during the 2017 season. Canopy eddy covariance observations were collected from a 10 m instrumented flux tower and reported at 30 minutes timestep. Leaf level Moni-PAM data were collected, using 5 emitter detector probes attached to the 4th plant leaf, at 10 minutes intervals and resampled/interpolated to 30 minutes intervals. As new leaves appeared, the probes were relocated to maintain their position on the 4th leaf from the top. Optical FloX measurements were collected from 1 m above the canopy, at the frequency defined by the system optimization program and were resampled/interpolated to the 30 minutes tower timestamps. The hight of the optics above the ground was adjusted to maintain the 1 m distance to the canopy during the growing season.
Data source location	Institution: The Optimizing Production inputs for Economic and Environmental Enhancement (OPE3) site at the Beltsville Agricultural Research Center (BARC) City/Town/Region: Beltsville, Maryland Country: United States of America Latitude and longitude (and GPS coordinates, if possible) for collected samples/data: 39.030686, 76.84546 Timeframe: 2017 growing season (June-October)
Data accessibility	Repository name: Mendeley Data Data identification number: http://dx.doi.org/10.17632/b84jk376c3.1 https://data.mendeley.com/datasets/b84jk376c3/draft?a=09b70ff8-599e-4405-a0f1-7a0c39e118fd
Related research articles	Campbell, P., K. Huemmrich, E. Middleton, et al. 2019. ``Diurnal and Seasonal Variations in Chlorophyll Fluorescence Associated with Photosynthesis at Leaf and Canopy Scales.'' *Remote Sensing*, 11 (5): 488 [10.3390/rs11050488] Yang, P., C. van der Tol, P. K. Campbell, and E. M. Middleton. 2020. ``Fluorescence Correction Vegetation Index (FCVI): A physically based reflectance index to separate physiological and non-physiological information in far-red sun-induced chlorophyll fluorescence.'' *Remote Sensing of Environment*, 240: 111676 [10.1016/j.rse.2020.111676] Yang, P., C. van der Tol, P. K. Campbell, and E. M. Middleton. 2021. ``Unraveling the physical and physiological basis for the solar- induced chlorophyll fluorescence and photosynthesis relationship using continuous leaf and canopy measurements of a corn crop.'' EGU *Biogeosciences*, 18 (2): 441-465 [10.5194/bg-18-441-2021]

1 The use of trade, firm, or corporation names in this article is for the information and convenience of the reader. Such use does not constitute official endorsement or approval by the US Department of Agriculture nor the Agricultural Research Service of any product or service to the exclusion of others that may be suitable.

## Value of the Data

• The data are useful and important for advancing the understanding of the links between the diurnal and seasonal dynamics of vegetation function, leaf chlorophyll fluorescence and canopy reflectance, vegetation indices (VIs), solar-induced fluorescence (SIF) and gross ecosystem productivity (GEP).
• The data can benefit ecologists, plant physiologists, foresters, and agriculturalists, by providing inputs for models and linking leaf and canopy processes to demonstrate the connections between reflectance and fluorescence properties for monitoring vegetation photosynthetic function and detecting stress.
• The data can be used by remote sensing professionals for calibration and validation of canopy VIs, reflectance and SIF signals currently measured by satellite instruments (e.g., DESIS/ISS, PRISMA/ASI, GOME-2, GOSAT, OCO-2, TROPOMI).
• The dataset is useful for simulations and generation of product prototypes characterizing photosynthetic function, as anticipated with space-based spectrometers, such as the existing DESIS (DLR, Germany) and PRISMA (Italy), and the forthcoming Surface Biology, Geology (SBG, NASA), EnMAP (DLR, Germany) and the European Space Agency (ESA) Fluorescence EXplorer (FLEX) mission, which will obtain globally canopy reflectance and SIF and will assess seasonal photosynthetic activity and canopy function.
• The fluorescence data might be used for improving the ability to scale and relate the commonly measured leaf-level plant physiology parameters (i.e., active chlorophyll fluorescence metrics such as electron transport rate, ETR and yield to photosystem II, PSII) to the canopy level solar induced fluorescence (SIF) measurements, which are targeted by the future NASA GeoCarb geostationary mission and ESA FLEX mission.

## 1 Data Description

There is a critical need for temporally dense time series of remote sensing data, collected at the ‘right’ time of day, frequency and season for monitoring the dymanics in vegetation function. To advance the ability to monitor the parameters governing vegetation function, time series capturing diurnal responses and seasonal changes in plant photosynthesis at leaf and canopy scales are needed. Leaf *in situ* and canopy proximal remote sensing can now provide such datasets for monitoring vegetation function at the temporal and spatial scales relevant to the dynamics of plant photosynthesis.

The dataset provided with this manuscript is comprised of tables with contemporaneous diurnal measurements collected continuously by three instruments (Table 1), measuring multiple maize leaves and the canopy during the 2017 growing season. Both raw instrument readings and the filtered and collated dataset are provided. The data are organized in comma delimited text files and excel files and are coupled by date and time of collection. Each excel file contains a ‘read_me’ sheet with information describing the specific parameters and processing approach. The raw data are provided for established users of the instrumentation and includes the separate records of each instrument, as follows:

• Moni-PAM data files:
  ○ MoniPAM_OPE3_2017_WinControl3.zip containing raw instrument readings. The files can be processed using the freely available WinControl3 software (https://www.walz.com/products/light/ulm-500/wincontrol-3.html, Heinz Walz GmbH).
  ○ MoniPAM2017_all.xlsx containins all readings, organised by time and date into one file. The file contains read_me sheet with definitions and units for all parameters.
• FloX optical data files*:*
  ○ FloX_DataOPE3_2017_raw.zip contains all FloX raw instrument readings. The data are organised in folders by date.
  ○ FLOX_R_OPE3_2017all.xlsx contains canopy diurnal reflectance measurements in the 400–850 nm region collected across the 2017 season.

**Table 1:** Combined dataset, including diurnal and seasonal observations of leaf and canopy fluorescence, canopy reflectance and photosynthetic CO_2_ dynamics for maize (*Zea mays* L.). The data are organized by date and time of collection (EST day-light savings/ UTC -4) in the Excel file 2017OPE3_Moni_FLOX_FLUX_ALL.xlsx.

Measurement type	Instrument ordata source	Dataset categories and data examples*
Canopy diurnal	FLoX	400-850 nm Reflectance
		SIF A and SIF B
		Reflectance VIs: NDVI, PRI, Chl_re,_ MTCI, etc.
	FLUX tower	H, LE, CO_2_ flux, Ta_4m, AR_in_LI190, PAR_out_LI190, Re, NEE, GEP, etc.
Leaf diurnal	Moni-PAM	Ft, Fo, Fm, Fm’, PAR, YII, ETR, NPQ, etc.

⁎ All parameters are defined in the ‘read_me’ spreadsheets in the excel files containing the data. The parameters listed in the categories above are provided in the combined dataset and listed in the appendix Table A1.

The collated dataset containing corresponding measurements from the three instruments (Table 1) is assembled in the excel file 2017OPE3_Moni_FLOX_FLUX_ALL.xlsx. Table 1 lists some of the parameters, which include: leaf active chlorophyll fluorescence parameters, canopy solar induced fluorescence (SIF), canopy reflectance and canopy eddy covariance measurements of photosynthetic function. The excel file 2017OPE3_Moni_FLOX_FLUX_ALL.xlsx contains a ‘read_me’ sheet with definitions and units for each parameter, which are provided also in Appendix Table A1.

• Terms and acronyms used in the manuscript and dataset:
 aPAR – absorbed PAR; ASCII - American Standard Code for Information Interchange; Clre – red-edge chlorophyll VI; CSV - comma delimited files; EDT – (UTC – 4); ETR - electron transport rate; F – light-adapted leaf chlorophyll fluorescence measured using the PAM approach (Ft – transient F, Fm’ – maximum F); faPAR – fraction of aPAR; FLEX - ESA Fluorescence EXplorer; FloX – automated dual spirometer system for measuring canopy radiance (Dual FLuorescence boX, JB Hyperspectral Devices UG, Dusseldorf, Germany); Gross ecosystem productivity (GEP); iFLD - Fraunhofer Line Discriminator method; Moni-PAM – automated PAM Data Acquisition system (MONI-DA, Heinz Walz GmbH, Effeltrich, Germany); *in situ* – measured under natural growing conditions in the field; NDVI – normalized difference VI; NPQ - non-photochemical quenching; OPE3 - the Optimizing Production inputs for Economic and Environmental Enhancement (OPE3) site at the Beltsville Agricultural Research Center, Beltsville, MD, USA; PAM – pulse amplitude modulated approach for measuring leaf chlorophyll fluorescence; PAR - photosynthetically active radiation; PRI – photochemical reflectance index; SBG - NASA Surface Biology and Geology mission; SFM - Spectral Fitting Method; SIF – solar induce fluorescence; SIF and reflectance; VI – vegetation reflectance index; YII/PSII - photochemical efficiency of photosystem II.

## 2 Experimental Design, Materials and Methods

### 2.1 Study site

The data were collected in 2017 at the Optimizing Production inputs for Economic and Environmental Enhancement (OPE3) site at the Beltsville Agricultural Research Center (BARC) on rain-fed maize (*Zea mays* L.). The OPE3 site is one of the Long Term Agro-ecosystem Research (LTAR) network of sites, operated by the US Department of Agriculture (USDA) Agricultural Research Service (ARS). It is a 22 ha production field located in Beltsville, Maryland, USA (lat/lon: 39.030686/-76.84546, 42 m asl). At OPE3 a 10 m tall flux tower is set up in a rainfed maize field, which is planted annually and maintained under optimal nitrogen treatment. The local climate is warm and temperate, with hot, humid summers, long fall and typically mild winters with occasional freezes, which provide a strong variation in seasonal leaf area index and canopy chlorophyll and biomass patterns.

### 2.2 Leaf-level measurements

Leaf-level fluorescence and photosynthetic efficiency measurements were collected continuously using the pulse amplitude modulated (PAM) approach, with an automated MoniPAM Data Acquisition system (MONI-DA, Heinz Walz GmbH, Effeltrich, Germany) outfitted with five emitter-detector probes. Leaf fluorescence, collected using the PAM approach, can provide the means for assessment of photosystem II (PSII) efficiency, which are highly dependent on the ambient light levels and can vary substantially with a relatively small change in photosynthetically active radiation (PAR) [6].

Five MoniPAM emitter-detector probes were mounted on five representative plants on the fully developed 4th leaf from the top of the plant. Measurement collection started when the corn canopy was well-established (e.g., 80-85% canopy closure) and continued until the end of the growing season. All readings are provied in the compressed archive MoniPAM_OPE3_2017_WinControl3.zip, which can be re-processed using the WinCintrol3 free software, available for download at https://www.walz.com/products/chl_p700/monitoring-pam/downloads.html. Three probes were positioned to measure fully sun-lit leaves, while two probes collected measurements on shaded leaves at varying illumination levels within the canopy.

The chlorophyll fluorescence (F) parameters measured directly include light adapted maximim and transient/steady-state flouorescence (Fm’ and F), and also the prohosynthetically active (PAR) excitation levels for each probe. From the direct measurements were derived F yield, photochemical efficiency of photosystem II (YII, or yield to PSII under steady state light), and electron transport rate (e.g., ETR, photochemical transport of electrons through PSII), which are used for characterising the photosynthetic activity of the plants.

### 2.3 Canopy-level measurements

Canopy diurnal reflectance and solar induced fluorescence measurements (SIF, mW/m^2^/nm/sr), both SIF_B_ (in the atmospheric O_2_B) and SIF_A_ (in the atmospheric O_2_A) bands, were collected using an automated, field dual-spectrometer system, the FLoX (Dual FLuorescence boX; JB Hyperspectral Devices UG, Dusseldorf, Germany) [1]. The FLoX down-welling optics were mounted at the top of a portable platform at approximately 3 m height. The upwelling optics were positioned at nadir and maintained at 1.5 m above the canopy throughout the growing season (i.e., by lifting periodically the measurement arm as new leaves developed and the canopy grew taller), viewing a 25° field of view.

The OPE3 site is instrumented with a 10 m eddy-covariance flux tower, which measures canopy level CO_2_ assimilation reported at 30 minutes intervals continuously throughout the growing season. The measured net CO_2_ flux (i.e., Net Ecosystem Exchange, NEE) is partitioned into gross primary productivity (GPP, the carbon used by photosynthesis) and ecosystem respiration [4,5].

## 3 Data Stratification and Processing

The maize crop conditions and phenology development in 2017 are recorded by the phenocam network (site arsope3ltar, data available at https://phenocam.sr.unh.edu/webcam/sites/arsope3ltar/). By DOY 190 the maize canopy was established, canopy closure was approximately 85% and no additional agricultural treatments were planned [7]. Measurements were collected continuously during the 2017 growing season, periodically re-locating the Moni-PAM probes, as new leaves emerged and lifting higher the FloX optics as the canopy grew taller. To enable analysis of the seasonal variation in the observations depending upon the time of collection and crop growth stage, the data were stratified into four growth stages*,* as follows: young (Yn, DOY 192-209); mature (M, DOY 220-235); senescent (DOY 236-272) which was subdivided into early senescence (S1, DOY 245-247) and advanced senescence (S2, DOY 255-257). The diurnal measurements were processed to form categorical variables representative of the three distinct periods during the day: morning (AM, 8:45-10:15), noon (11:45-1:15) and afternoon (PM, 15:15-16:45) local time (EDT or UTC-4).

Leaf-level light-adapted PAM fluorescence metrics representative of the canopy were derived by calculating the mean values from the three Moni-PAM emitter-detector probes maintained on fully sunlit leaves, using the simultaneously acquired measurements at 10 minutes interval. Large outlier values from a single probe were removed. Average values for light adapted fluorescence (F, relative units), mean yield of Photosystem II (YII), mean relative electron transport rate (ETR) and PAR were calculated. The night-time measurements were used to derive Fo and Fm, and to calculate non-photochemical quenching (NPQ), which are described in [8] and provided in file MoniPAM2017_all.xlsx.

The FLoX system collects upwelling and downwelling measurements, which were processed to reflectance and solar induced fluorescence (SIF) using two open-source R packages [2,3] (i.e., FieldSpectroscopyDP and FieldSpectroscopyCC), which are available for download at the following links: https://github.com/tommasojulitta/FieldSpectroscopyDP and https://github.com/tommasojulitta/FieldSpectroscopyCC. Quality screening of the measurements was completed using the assigned by the R routines quality flags, which reported information related to the illumination stability during the measurement cycle and the internal noise of the instrument during data acquisition. The dataset was filtered for saturated data points and anomalous readings. Additional screening was done to remove sensor artifacts due to low light levels (i.e., in the early morning and late afternoon, where the solar zenith angle, SZA is >75^o^). The outputs of the processing include incoming radiance at the surface; top of canopy reflected radiance; apparent reflectance; and SIF estimates, at spectral wavelengths associated with both atmospheric oxygen absorption features centered at 683 (SIF_B_) and 760 nm (SIF_A_). SIF_A_ and SIF_B_ were retrieved by applying the Fraunhofer Line Discriminator method (version 3, iFLD) and the Spectral Fitting Method (SFM) [9,10]. Total SIF_A+B_ was calculated as the sum of SIF_A_ and SIF_B_ [7]. Canopy reflectance from the FLoX was used to calculate indices indicative of vegetation green biomass, chlorophyll content and photosynthetic function (e.g., NDVI, Chl_re_ and PRI), which are described by providing the formulas for their calculation in the ‘read_me’ sheets of the excel files containing the data.

Incident canopy PAR was measured by the OPE3 flux tower and the FloX instrument. Incident PAR was measured at leaf level by the Moni-PAM emitter-detector probes. Outliers (e.g., values greater than three standard deviations from the mean) from the daily linear trend were removed from all data sets. The PAR absorbed by the canopy (APAR, µmol/m^2^/s) in the FLoX footprint is required for computation of the FLoX fluorescence yields. It was calculated as: APAR = PAR* *f*APAR, where *f*APAR, the fraction of incident PAR absorbed by the canopy, is estimated using an equation available for OPE3 from [11]. Canopy SIF yield (YSIF) was calculated as: YSIF = SIF/APAR (for both SIF A and SIF B bands, with the assumption that APAR was equally available to both PSII and PSI.

The set of contemporaneous complementary leaf and canopy measurements at OPE3 were collected at different time intervals: 30 minutes (flux tower), 10 minutes (Moni-PAM), and at the time required for system optimization (i.e., approximately 1 minute, FLoX). To compare all three data sets, the most frequently acquired FLoX data set was screened for outliers and smoothed to reduce the random noise using a moving Savitzky-Golay filter function (MATLAB 2019) and linearly interpolated to 10 minutes intervals and resampled at congruent time steps with the Moni-PAM data. The combined Moni-PAM and FLoX data were then linearly interpolated to extract values at the same 30 minutes intervals used by the flux data.

## Ethics Statement

The work did not involve the use of human subjects, animal experiments and information collected from social media platforms.

## CRediT authorship contribution statement

**Petya Campbell:** Conceptualization, Methodology, Software, Data curation, Formal analysis, Writing – review & editing. **Elizabeth Middleton:** Conceptualization, Methodology, Formal analysis, Writing – review & editing. **Karl Huemmrich:** Conceptualization, Methodology, Formal analysis, Writing – review & editing. **Lauren Ward:** Software, Data curation, Formal analysis. **Tommaso Julitta:** Conceptualization, Software, Data curation, Formal analysis, Writing – review & editing. **Peiqi Yang:** Software, Data curation, Formal analysis. **Christiaan van der Tol:** Formal analysis. **Craig Daughtry:** Formal analysis. **Andrew Russ:** Software. **Joseph Alfieri:** Software, Data curation, Formal analysis. **William Kustas:** Methodology, Formal analysis.

## Declaration of Competing Interest

The authors declare that they have no known competing financial interests or relationships which have or could be perceived to have influenced the work reported in this article.

## Figures and Tables

**Table:** 

Variable name	Description	Units
DOY	Day of year	days
DateTime(EST/DLS)	Local dayllight savings time = Eastern standard Time −1 hr	dd-mm-yyyy hh:mm:ss
Month	Month of observation	months
Date	Date of observation	dates
GS	Growth Stages	periods: young, mature, early senescence, advanced senescence
Time	Time (hh:mm)	hours and minutes
3TGs	time grouping in 3 groups: morning, mid-day, afternoon	am, noon, pm

*Moni-PAM data variable*		

AvgF	Average Ft (F transient) of 3 sensors in a sunlit portion of the canopy	ru
AvgFm	Average Fm (Fmax) of 3 sensors in a sunlit portion of the canopy	ru
AvgPAR	Average PAR of 3 sensors in a sunlit portion of the canopy	µmol/m^2^/s
AvgTemp	Average temperature of 3 sensors in a sunlit portion of the canopy	T° C
AvgYII	Average yield to photosystem II (YII) of 3 sensors in a sunlit portion of the canopy	ru
AvgETR	Average election transport rate (ETR) of 3 sensors in a sunlit portion of the canopy	ru

*FLoX data variables*		

Lat	Latitude of observation	degrees
Lon	Longitude of observation	degrees
SZA	Solar zenith angle at the time of observation	degrees
Inc750	Incoming radiance at 750 nm	W/m2/nm/sr
Refl760	Reflected radiance at 760 nm	W/m2/nm/sr
Refl687	Reflected radiance at 687 nm	W/m2/nm/sr
Refn750	Reflectance at 750 nm	0-1
Refn760	Reflectance at 760 nm	0-1
E_stability	Sun stability during the measurement	[%]
SIF_A_ifld	SIF A calculated using the ifld approach	mW/m2/nm/sr
SIF_B_ifld	SIF B calculated using the ifld approach	mW/m2/nm/sr
SIF_B/A_ifld	SIF_B_ifld/SIF_A_ifld	ratio
SIF_A_sfm	SIF A calculated using the spectral fitting method	mW/m2/nm/sr
SIF_B_sfm	SIF B calculated using the spectral fitting method	mW/m2/nm/sr
SIF_B/A_sfm	SIF_B_sfm/SIF_A_sfm	ratio
*Reflectance vegetation indices*	*Formula (R Index;wl;fwhm;expr;convolution)*	
NDVI	NDVI;``780;680'';``10;10'';(a-b)/(a+b);mean	ratio
PRI	PRI;``531;570'';``2;2'';(a-b)/(a+b);mean	ratio
MTCl	MTCI;``754;709;681'';``5;5;5'';(a-b)/(b+c);mean	ratio
SR	SR;``830;665'';``20;30'';a/b;mean	ratio
EVI	EVI;``830;665;490'';``20;30;30'';2.5*(a-b)/(a+6*b-7.5*c+1);mean	ratio
REP	REP;``665;830;705;740'';``30;20;15;15''; 705+35*((a+b/2)-c)/(d-c);mean	ratio
TCARI	TCARI;``705;665;560'';``15;30;35'';(3*(a-b)-0.2*(a-c)*(a/b));mean	ratio
REDCl	REDCL;``830;705'';``15;15'';(a/b-1);mean	ratio
MCRI	MCARI;``705;665;560'';``15;30;30'';((a-b)-0.2*(a-c))*(a/b);mean	ratio
EPAR	upwelling PAR	quality parameter
LPAR	downwelling PAR	quality parameter
PARi_from750	proxy of PARi	mW/m2/nm/sr
faPAR	Empirical model	available for OPE3 from [11]
aPARi	PARi*faPAR	ratio
*SIF vegetation indices*	*Formula*	
SIFAifld/PARi	SIF_A_ifld / PARi	ratio
YSIFAifld/aPARi	YSIF_A_ifld / aPARi	ratio
SIFBifld/PARi	SIF_B_ifld / PARi	ratio
YSIFBifld/aPARi	YSIF_B_ifld / aPARi	ratio
YSIFifld_A+B	(SIF_A_ifld + SIF_B_ifld)/aPARi	ratio
SIFAsfm/PAR	SIF_A_sfm / PARi	ratio
YSIFAsfm/aPAR	YSIF_A_sfm / aPARi	ratio
SIFBsfm/PAR	SIF_B_sfm / PARi	ratio
YSIFBsfm/aPAR	YSIF_B_sfm / aPARi	ratio
YSIFsfm_A+B	(SIF_A_sfm + SIF_B_sfm)/aPARi	ratio

*Eddy covariance data*		

Rn_CNR1	Net radiation	(W/m^2^)
G_total	Soil heat flux	(W/m^2^)
H	Sensible heat flux	(W/m^2^)
LE	Latent heat flux	(W/m^2^)
CO2_flux	CO2 flux	mg/(m^2^ s)
U_sonic	Wind speed from sonic anemometer	(m/s)
Ustar_sonic	Friction velocity	(m/s)
WD_sonic	Wind direction from sonic anemometer	(degrees)
Ta_4m	Air temperature at 4 m	(C)
VP_4m	Vapor pressure at 4 m	(kPa)
PAR_in_LI190	Incident PAR	umol/(m^2^ s)
PAR_Out_LI190	reflected PAR	umol/(m^2^ s)
Rs_in_CNR1	Incident shortwave radiation	(W/m^2^)
Rs_out_CNR1	reflected shortwave radiation	(W/m^2^)
Precip	Precipitation	(mm)
Closure ratio	quality parameter	ratio
Re	Calculated ecosystem respiration	mg/(m^2^ s)
GEE	Calculated gross ecosystem exchange (CO2 into canopy)	mg/(m^2^ s)
GEP	Calculated gross ecosystem production (-GEE)	mg/(m^2^ s)
GEP/aPAR	light use efficiency	

## Appendix

**Table A1**Variables name/acronym, description and unit for the derived variables available in the in the combined/collated dataset provided in file 2017OPE3_Moni_FLOX_FLUX_ALL.xlsx.
